# Spatial Accessibility to Primary Healthcare Services by Multimodal Means of Travel: Synthesis and Case Study in the City of Calgary

**DOI:** 10.3390/ijerph16020170

**Published:** 2019-01-09

**Authors:** Amritpal Kaur Khakh, Victoria Fast, Rizwan Shahid

**Affiliations:** 1 Department of Geography, University of Calgary, Calgary, AB T2N 1N4, Canada; amritpal.khakh@ucalgary.ca; 2 Primary Health Care, Alberta Health Services, Calgary, AB T2W 3N2, Canada; Rizwan.shahid@ahs.ca

**Keywords:** spatial accessibility, multimodal network, primary healthcare

## Abstract

Universal access to primary healthcare facilities is a driving goal of healthcare organizations. Despite Canada’s universal access to primary healthcare status, spatial accessibility to healthcare facilities is still an issue of concern due to the non-uniform distribution of primary healthcare facilities and population over space—leading to spatial inequity in the healthcare sector. Spatial inequity is further magnified when health-related accessibility studies are analyzed on the assumption of universal car access. To overcome car-centric studies of healthcare access, this study compares different travel modes—driving, public transit, and walking—to simulate the multi-modal access to primary healthcare services in the City of Calgary, Canada. Improving on floating catchment area methods, spatial accessibility was calculated based on the Spatial Access Ratio method, which takes into consideration the provider-to-population status of the region. The analysis revealed that, in the City of Calgary, spatial accessibility to the primary healthcare services is the highest for the people with an access to a car, and is significantly lower with multimodal (bus transit and train) means despite being a large urban centre. The social inequity issue raised from this analysis can be resolved by improving the city’s pedestrian infrastructure, public transportation, and construction of new clinics in regions of low accessibility.

## 1. Introduction

Internationally, access to primary healthcare services (e.g., family doctor) has long been widely accepted as one of the primary goals in fulfilling the health needs of individuals since these are often the first point of contact in the healthcare system; providing a wide range of services over time that focus on prevention and prognosis of diseases through early diagnosis, contrary to disease-oriented care [1,2,3,4]. Statistically, as of 2016, Canada has 2.6 physicians per 1000 people, which is significantly lower than the Organization for Economic Co-operation and Development (OECD) countries’ average of 3.3 physicians per 1000 population [5]. Lower physician availability status in Canada, compared to the international standards, is escalated by uneven distribution of population and healthcare facilities over regions. As such, access to primary healthcare continues to be a pressing research and policy issue in Canada and globally.

Despite this, the term ‘access’ is still not well-defined [6,7,8,9,10]. The reason behind the ambiguity in defining healthcare access is that it is a multidimensional term. Access can be defined both as a noun, referring to the potential for healthcare use; and, a verb, referring to the interaction between the provider and the patient [3,11]. In order to better interpret access, it has been presented in terms of stages and dimensions. The two stages are ‘potential’ for healthcare and ‘realized’ service utilization, which correspond to the noun and verb definitions, respectively, of access [3].

The progression from potential to realized access can be impeded by the presence of a number of barriers. Penchansky and Thomas (1981) group the barriers according to five dimensions: availability, accessibility, affordability, acceptability, and accommodation. The last three aforementioned dimensions comprise the aspatial factors (independent of any geographic aspect), and refer to healthcare costs, cultural attributes and communication effectiveness, respectively. The first two dimensions (availability and accessibility) contain an inherent spatial component where the former refers to the capacity of the provider and the latter refers to the travel cost between the provider and the patient [12]. Commonly, in urban areas, where there is a provision of multiple provider service locations, availability and accessibility dimensions of access are considered in coherence. This union is referred to as ‘spatial accessibility’ [3].

Multiple methods, and combinations of methods, have been developed to derive an effective spatial accessibility measure, including traditional measures such as straight distance and supply-to-demand ratio [13,14], and advanced measures, such as two-step floating catchment area [15], three-step floating catchment area [10,16], and kernel density and enhanced variable two-step floating catchment area method (EV2SFCA and KD2SFCA) [14,17], with many others still actively under development by the research community. These measures of spatial accessibility have been extensively applied to detect non-uniform distribution of healthcare [10,16,18,19,20,21,22], and to optimize access to other services such as daycares, libraries, food stores, and district building energy plan [13,23,24]. Despite this growth in spatial accessibility methods, there is limited research spatial accessibility to primary healthcare by mode of transit.

When it comes to spatial access to primary healthcare, the assumption that all populations have access to a car which enables them to access the primary healthcare is highly generalized in the current measures. Car ownership is not universal. In fact, fewer people are getting their driver’s license in the global south and north due to improved public and active transportation. A study led by the University of Michigan Transportation Research Institute showed that the number of young licensed drivers has decreased in half of the 15 countries they investigated, including Canada. Specifically, between the ages of 25 to 34, 92% percent of the people had a driver’s license in 1999 and in 2009, this number dropped to 87% [25]. Further to this point, cities are encouraging citizens to employ greater use of active transportation networks, where an effective transition requires service and amenities that are accessible—by transit, and/or walking. To more accurately understand spatial access for non-drivers, it is crucial to measure the accessibility to primary healthcare facilities by alternative modes of travel.

Collectively, the distribution of facilities and the spatial networks of transportation are two significant determinants of spatial accessibility of primary healthcare facilities. Realizing the importance of adequate primary healthcare and mode of transportation, this research analyzes multimodal access to primary care in the City of Calgary, Canada. First, we explore current methods of measuring access, before settling on the spatial access ratio (SPAR) method. Then, spatial accessibility to the primary healthcare facilities in the city is analyzed, using SPAR, at the community level by simulating travel on walking, multimodal, and driving-oriented networks.

## 2. Data and Methods

### 2.1. Current Methods for Measuring Access

Over the last decades, various methods have been developed and applied to measuring spatial access. Guagliardo [3] has broadly grouped accessibility measures into four different categories: provider-to-population ratio, distance to the nearest provider, average distance to a group of providers, and gravity models. The first three metrics are easy to implement. With the advancement of Geographic Information Systems (GIS), access measures and conceptualizations have evolved to incorporate spatial measurement, a fundamental component in gravity models. The gravity models are considered to encompass the interaction between the provider and population more accurately than the methods in other three categories [26].

The common measure considered in spatial accessibility to primary healthcare facilities is Two Step Floating Catchment Area (2SFCA), a derivative of gravity models [17,21]. Similar to gravity models, these measures account for both supply (capacity of provider), demand (population needs) and the distance impedance components of spatial accessibility [16,18,27]. However, Luo and Wang [18] recognize two limitations to their 2SFCA methodology. Firstly, by considering a threshold travel time, it is concluded that 2SFCA provides a dichotomous measure (accessible/not accessible). In other words, the locations outside the catchment area are considered to have no access, whereas the resident catchment with service locations falling inside are considered to have full access to the services, due to the presence of artificially sharp catchment boundaries. Secondly, the distance decay within the catchment is not considered, it is assumed that all population locations within this area have an equal access to the primary care facility [18].

Attempts have been made to overcome the limitations of 2SFCA. One of the significant barriers recognized in access to healthcare is distance impedance between an individual’s location and the primary healthcare facility. The distance impedance within the catchments of 2SFCA has been ignored which is usually not the case in the real world. As the distance increases, people tend to utilize the distant services less than the services available nearby [28]. The advanced catchment area methods have been synthesized where researchers have proposed putting weights within the catchment areas, incorporating continuous Gaussian distance decay, adjusting the catchment areas to meet different provider-to-provider ratio thresholds [29,30,31]. Even though efforts have been made to reflect distance decay measure and to adjust supply and demand in the Floating Catchment Area methods accurately, the applicability of these models is still limited due to inaccuracies which may have been introduced in the analysis by choosing arbitrary distance friction coefficient values [32].

The optimal way to infer an accurate distance coefficient value is to analyze the previous provider–patient interaction records, such as the time it takes the patient to access healthcare services. However, data on provider–patient interaction are not readily available which restricts the researchers to model the impedance by using the arbitrary friction values. Wan et al. [32] have proposed a measure, referred to as Spatial Access Ratio (SPAR) to overcome the uncertainty issues arising from previous accessibility measures. This measure is an advancement over the Enhanced 2SFCA method because it is less sensitive to distance impendence variable than the aforementioned methods. Ultimately, we chose to employ the SPAR method in this study.

Next, to more accurately understand spatial access for non-drivers, it is crucial to measure the spatial accessibility to primary healthcare by alternative modes of travel—considering walking and transit modes. Mao and Nekorchuk [33] have simulated the multimodal approach by specifying incremental catchment areas for different travel modes and obtaining the sum of the populations reached through different modes from healthcare facilities. However, this methodology has a limitation in the nature of the dataset used. To model travel by bus and car, the authors used the road network in simulating travel whereas the walkable mode of travel was not included in the multimodal analysis, concluding that doing so might have resulted in less heterogeneity in their results since people may prefer to access facilities in close proximity via the sidewalk or trail network.

In assessing walkability, it is often assumed that the roads selected to model pedestrian behavior are lined with sidewalks in a real setting. These results might lead to an overestimation of accessibility measures in regions where there is no proper sidewalk infrastructure. Iacono et al. [34] have pointed that the network specifications for walking are different than driving and are required to be presented at a finer scale due to different dimensions of the infrastructure. Using roads as a network for walking can result in loss of resolution since the road network cannot identify many of the shorter trips made by walking, and eventually, this results in uncertainty in the outputs [34]. Aimed towards bridging the gap between the travel networks used in access studies, this research focuses on measuring the access to primary healthcare in Calgary by considering appropriate network infrastructures. Specifically, sidewalks, trails and pathways were integrated into the analysis to model walking; bus routes and train network to model public transportation; and road networks to model driving.

### 2.2. Study Area

**Figure 1:**
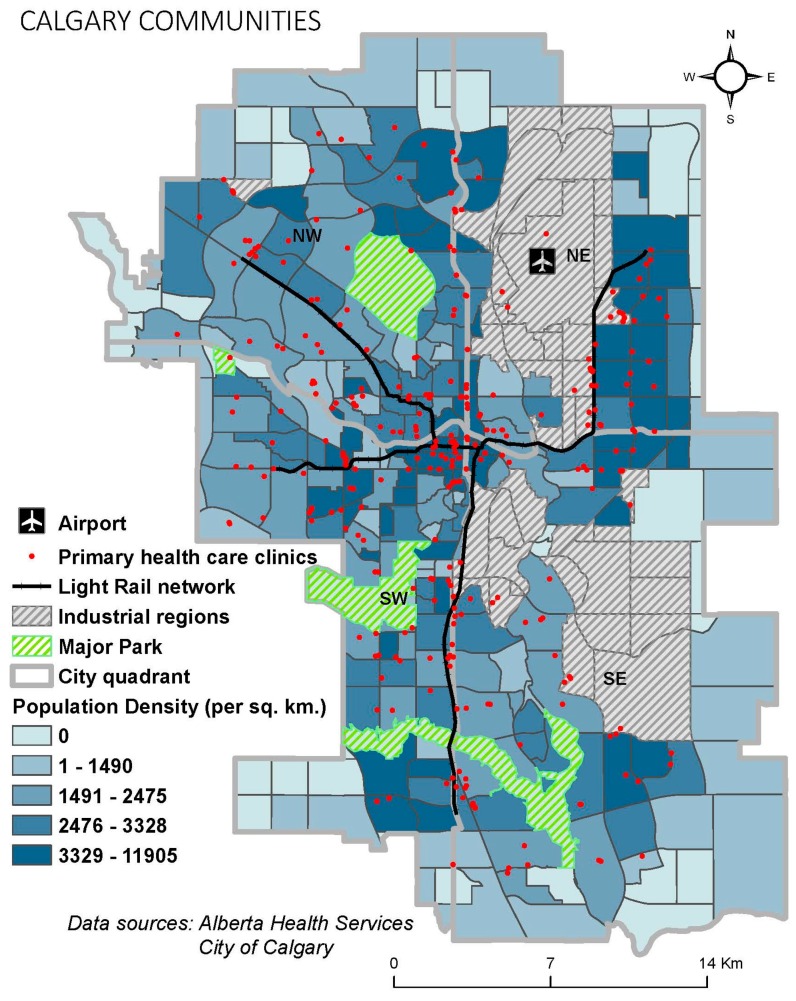
City of Calgary—Primary Healthcare Services by Community.

**Figure 2:**
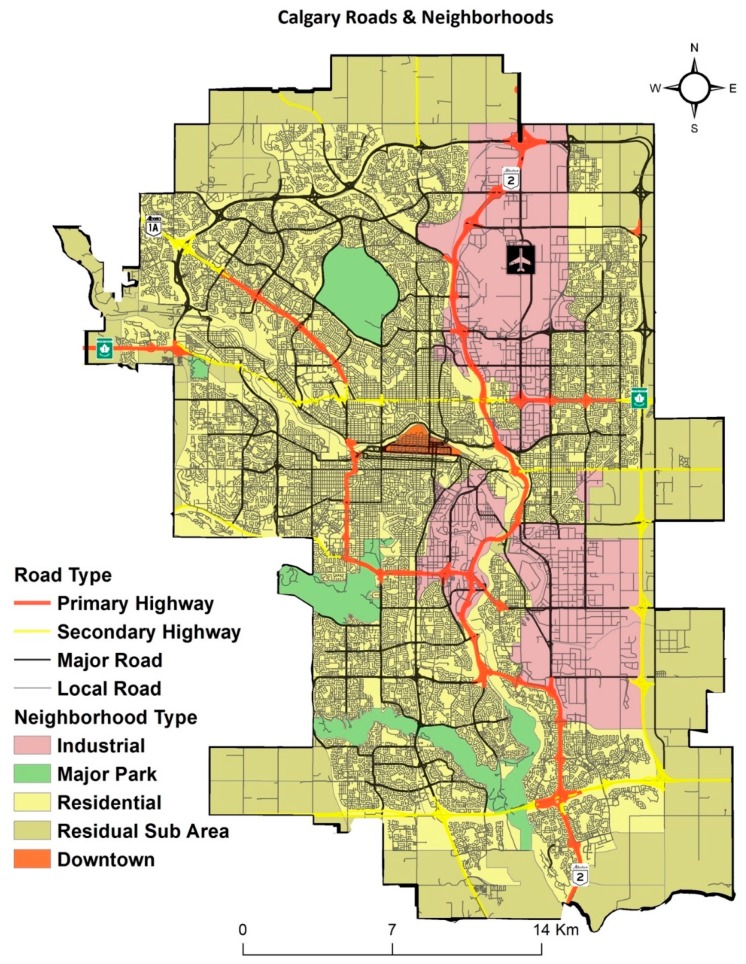
City of Calgary—Roads and Neighborhoods.

This study aimed to calculate the spatial accessibility of primary healthcare facilities by different travel modes in the city of Calgary in the province of Alberta, Canada. As of 2016, the City of Calgary had a total population of 1,239,220 (census subdivision), a land area of 5110 square kilometers, and a population density of 273 people per square km [35]. Administratively, there are 198 different communities (Figure 1) and 1594 dissemination areas (DA) in Calgary. Calgary also contains large urban parks and industrial regions that contain zero population. While the analysis was performed on all neighborhood types (see Figure 2), we show the areas with a hatched symbology to accurately reflect areas with no access versus areas with no population.

There is a physician shortage in Canada overall, and even more so in the province of Alberta and City of Calgary. In 2014, Statistics Canada reported that 14.9% of Canadians of age 12 and older did not have a regular family physician, whereas in Alberta, this percent increases to 19.9% [36] Within Alberta, 80.2% of people in the City of Calgary had an access to a family doctor as compared to 81.1% in the City of Edmonton. Furthermore, in Calgary, the cost of a standard hospital stay between 2015 and 2016 was calculated to be $8233, which is higher than the figures at national ($6098) and provincial ($8007) levels [37]. The lower than average access to a primary care physician in Alberta and specifically, in the City of Calgary, with high hospitalization cost leads to an extensive financial, mental, and physical toll on the population; emphasizing the importance of spatial accessibility to primary healthcare status in the city.

The analysis was performed at the dissemination area (DA) level as this is the smallest level of census division and consists of 400 to 700 people. This approach was taken to limit any inaccuracies resulting from loss of spatial resolution. On the other hand, the results are disseminated at the community level since, at this level, health statuses are derived based on various health parameters and city policies are set at community levels as people tend to associate themselves with the communities they are residing [38,39].

### 2.3. Data

The datasets used for this study are presented in Table 1. The clinic and sidewalk data were obtained directly from the source (the City of Calgary and Alberta Health Services respectively) and provide a snapshot of spatial accessibility at the time the analysis was performed. To make the sidewalk network more complete, the links between the sidewalks were generated by creating a buffer from the road intersections layer. This connected sidewalk data, along with trails and pathways were merged to model the walkable network. The walking speed was calculated as 4.8 km/h as per City of Calgary average walking speed standards [40,41]. While average walking speed does not consider different abilities, El-Geneidy el al. [42] compared different travel speeds used for walkability in literature and conclusively, proposed that the assumption of constant speed can be accepted to model travel by walking.

The catchment radius for the methodology is 30 min, as this is most widely used in literature to measure spatial accessibility to primary healthcare facilities [18,31]. With the above considerations, the simple conversion of distance to time was calculated for sidewalks, trails, and pathways data to model walking. The bus routes and road network data was also converted from distance to time in ArcGIS with the following speed limit considerations for different roads in the City of Calgary (Table 2).

### 2.4. Building Spatial Network by Mode of Transportation

Each mode of travel was simulated in GIS platform through ESRI’s ArcGIS (ESRI, Redlands, CA, USA) Network Analyst tool. The network analysis was performed on time instead of distance to facilitate comparison in results obtained by different modes of travel. Additionally, in past spatial accessibility studies, the catchment thresholds are also presented in time, specifically 30 min for the urban area [10,18,27,31]. For consistency, the same threshold time was set.

#### 2.4.1. Building a Road Network

The first mode of travel in the analysis—access by driving—was simulated by travel along the roads, consisting of primary highway, secondary highway, major road, and local road (Figure 2). The road-based analysis covers all car travel, including car ownership, taxis or newer shared mobility (i.e., Car2go) models. This dataset composed of end point connectivity between the road segments in the City, upon which topological relations were built as part of the road network analysis. Since the time attribute was required for the accessibility method to be applied, the conversion from distance to time was calculated with the speed limits specified in Table 2.

#### 2.4.2. Building a Sidewalk Network

**Figure 3:**
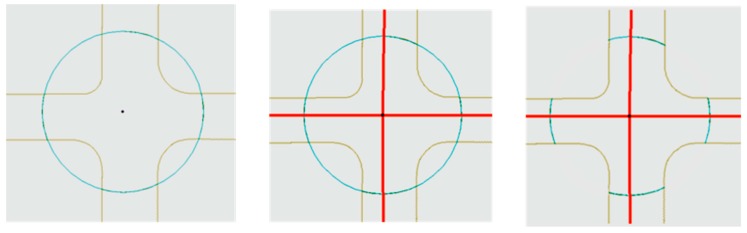
An illustration of analysis performed for crosswalk generation in ArcGIS (red lines = road network).

The sidewalk data obtained from the City of Calgary was not connected but rather, was presented in distinct gridded rectangular segments for each block. In order to create a functional walkable network, the following steps were completed: firstly, an 18-m radius buffer was created from the intersection point layer. The resulting buffer polygon was converted to line feature class and the lines were split at the points of intersections between the sidewalks and the line buffer. Finally, only those lines were retained which intersected with the road shapefile. This provided us with the crosswalks as the connections from one sidewalk block to another. One of the connections generated between sidewalks is presented in Figure 3. Eventually, the crosswalk and the sidewalk layers were merged together to create a walkable network. Considering the constant speed of travel (4.8 km/h), the simple conversion was performed to create walkable catchments based on travel time. This provided the second mode of travel in the analysis: accessibility by walking.

#### 2.4.3. Building a Multimodal Network: Sidewalks Plus Bus Routes and Train Lines

**Figure 4:**
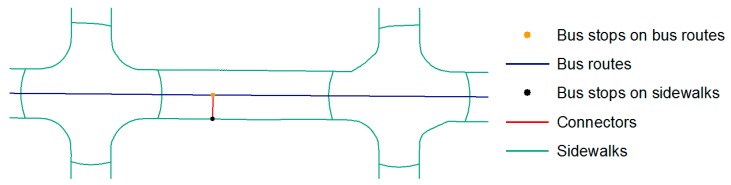
An illustration of connections between sidewalks and bus routes.

The third mode of travel—accessibility by multimodal network—was created to simulate travel by public transit. The multimodal network was created by combining walkable network layer, bus routes (trip time was determined by calculating the time between the first and the last stop in the bus routes), bus stops as the link between the sidewalks and the bus routes, train lines, and train stations as a link between the sidewalks and the trains. The travel speed for bus routes were determined according to the speed specifications in Table 2. The bus routes were split at each bus stop to provide the entry and the exit points to buses from the sidewalks (Figure 4). Eventually all aforementioned layers were combined to build a functional multimodal network.

### 2.5. Spatial Accessibility Calculations by Different Modes of Travel

The spatial accessibility ratios were calculated based on the Spatial Access Ratio (SPAR) described in Section 2.1. This methodology was applied individually—by walking, multi-modal, and driving modes—to compare accessibility to the primary healthcare facilities by different modes of travel. We began by applying the distance impedance variant of the Two-Step Floating Catchment Area method: Enhanced Two-Step Floating Catchment Area (E2SFCA) [31]. We presented this advancement over 2SCFA to address its uniform access problem within the catchment area. Similar to 2SFCA, this method is applied in two steps (supply and demand models) with the addition of Gaussian weights to introduce distance decay within catchments.

Step 1 (supply model): A 30-min service area was generated around each primary care facility. The catchment was further divided into three travel subzones: the first zone between 0–10 min, second zone between 10–20 min, and third zone between 20–30 min. The population locations contained within each subzone were identified and the population at these locations was weighted according to the subzone it was contained within. Next, the Provider-to-Population ratio, *Rj*, of the primary care facility is calculated based on Luo and Qi’s formula [31]:

$$R_{j}=\frac{S_{j}}{\sum_{k\in \left\{d_{kj}\leq D_{r}\right\}}P_{k}W_{r}}\;=\frac{S_{j}}{\sum_{k\in \left\{d_{kj}\leq D_{1}\right\}}P_{k}W_{1}+\sum_{k\in \left\{d_{kj}\leq D_{2}\right\}}P_{k}W_{2}+\sum_{k\in \left\{d_{kj}\leq D_{3}\right\}}P_{k}W_{3}}$$

where

$P_{k}$ refers to the population at the DA centroid location, *k*, falling within the catchment size *j*
$S_{j}$ refers to number of general practitioners at the facility *j*
$d_{kj}$ corresponds to the travel time between *k* and *j*
$D_{r}$ is the *r*th travel time zone (where *r* = 1, 2 or 3)

Step 2 (demand model): From every population centroid location, all the primary care facility locations are identified within its 30-min service area in ArcGIS. The Provider-to-Population ratio, *Rj*, for the identified facility locations within the location are summed (Lu and Qi, 2009):

$$A_{i}^{F}=\underset{j\in \left\{d_{ij}\in D_{r}\right\}}{\sum }R_{j}W_{r}\;=\underset{j\in \left\{d_{ij}\in D_{1}\right\}}{\sum }R_{j}W_{1}+\underset{j\in \left\{d_{ij}\in D_{2}\right\}}{\sum }R_{j}W_{2}+\underset{j\in \left\{d_{ij}\in D_{3}\right\}}{\sum }R_{j}W_{3}$$

where

$A_{i}^{F}$ denotes the accessibility of population at location, *i*, to the facility
$R_{j}$ corresponds to the weighted provider-to-population ratio (Step 1) that falls within the catchment size *i*
$d_{ij}$ represents the travel time between *i* and *j*
$W_{r}$ is the distance decay weight

The values of weights chosen for the three travel zones (1.00, 0.42 and 0.09 for *W*_1_, *W*_2_ and *W*_3_ respectively) represent the sharper distance decay, which is prominent in the case of the presence of multiple service facilities, such as in the urban context. The rationality for these weights is based on the assumption that people tend to travel lesser distance in the presence of choice between the services [31]. Specifically, we use the sharper distance decay weights—*W*_1_, *W*_2_, and *W*_3_ relating to the 0–10, 10–20, and 20–30 min range respectively—that Lou and Qi [31] establish for use in urban areas where there are more choices of facilities, rather than remote or rural areas where a slow distance decay is more realistic (people in rural areas travel further by necessity).

After applying the methodology of E2SFCA, the final step was to normalize the accessibility values as proposed by Wan et al. [43] to overcome the distance impedance uncertainty issues arising from previous accessibility measures. The SPAR values were calculated as a ratio between spatial accessibility index (that is, *A_i_^F^* at location, *i*) and the mean spatial accessibility of all population locations to derive normalized spatial accessibility indices. The SPAR methodology was applied in the GIS environment to analyze spatial accessibility to primary healthcare services by driving, walking, and multimodal (bus routes and train) means of travel from aforementioned networks. Quantile classification method was used to group the SPAR values with modifications to isolate the 0 values. Eventually, the shortage areas, exhibiting the 0 SPAR values, were identified and the population within the shortage areas was calculated by mode of travel.

### 2.6. Multiple Regression Analysis

Multiple regression analysis was performed using *R* to identify the relationship between accessibility indices by three modes of travel (SPAR values) and different social determinants of health, comprising of Pampalon Index (Table 3). The social determinants were comprised using the Pampalon Index (Table 3); a small area-based index used to reflect deprivation of relationships among individuals in workplace, community and family (social deprivation) and the deprivation of wealth, conveniences, and goods (material deprivation) [44,45].

These determinants, at the DA level, were analyzed for correlation to reduce any redundancy in the results. Non-correlated independent variables were included in the multiple regression models for the results obtained from the three modes of travel. Since it is a spatial accessibility analysis, the regression models were subjected to measure spatial autocorrelation. To identify spatial autocorrelation in the SPAR values, Moran’s I statistics were calculated for the accessibility values from three modes of travel. Due to the presence of spatial autocorrelation, spatial regression [46,47,48] was performed in *R* to account for spatial dependency, providing more accurate regression results.

For the spatial regression model, the predictor coefficients were obtained for the significant variables and their values were interpreted to deduce the link between the variables and the SPAR indices. The non-correlated variables for regression analysis were determined through correlation matrix analysis with a cut-off value of 0.70. The variables with the correlation values below 0.70 were retained in the regression analysis. Multiple regression analysis was performed on the selected independent variables with SPAR values as the dependent variable.

## 3. Results

**Figure 5:**
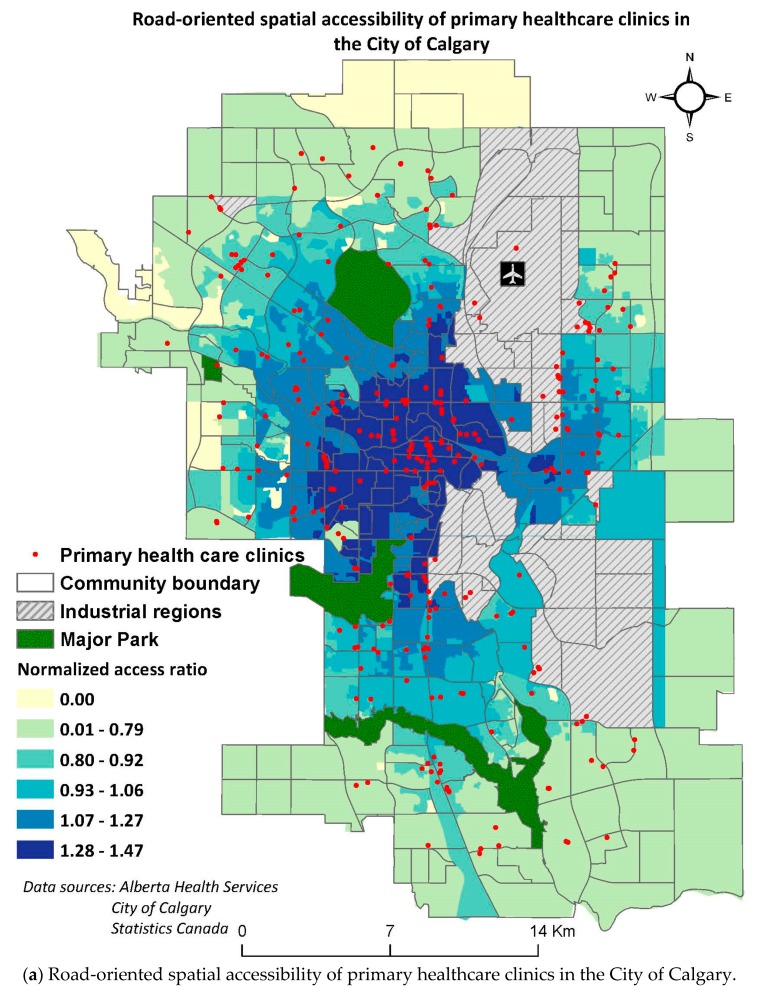

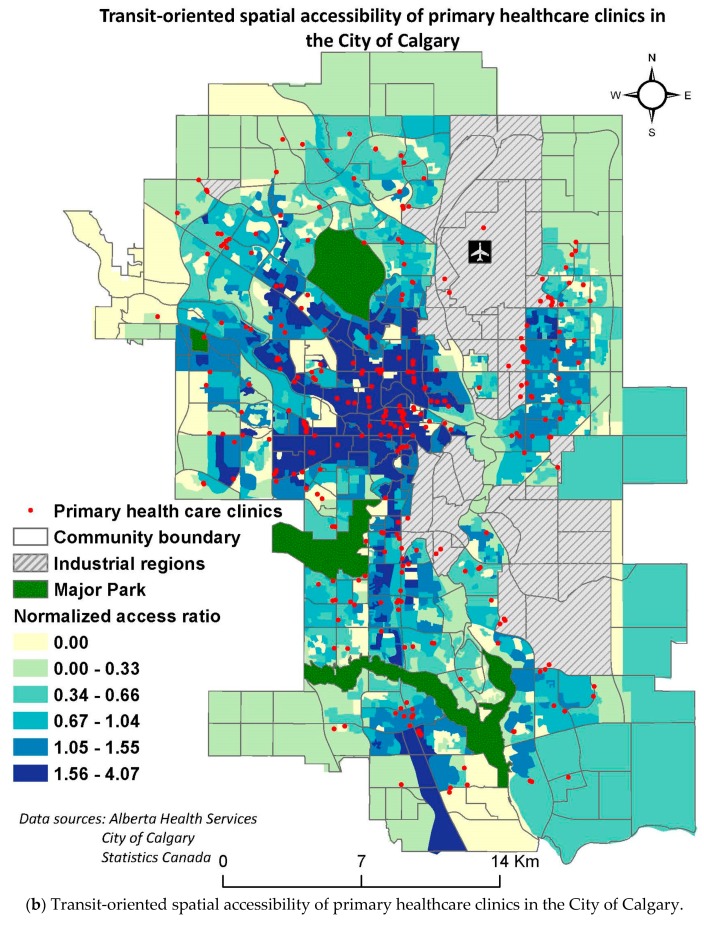

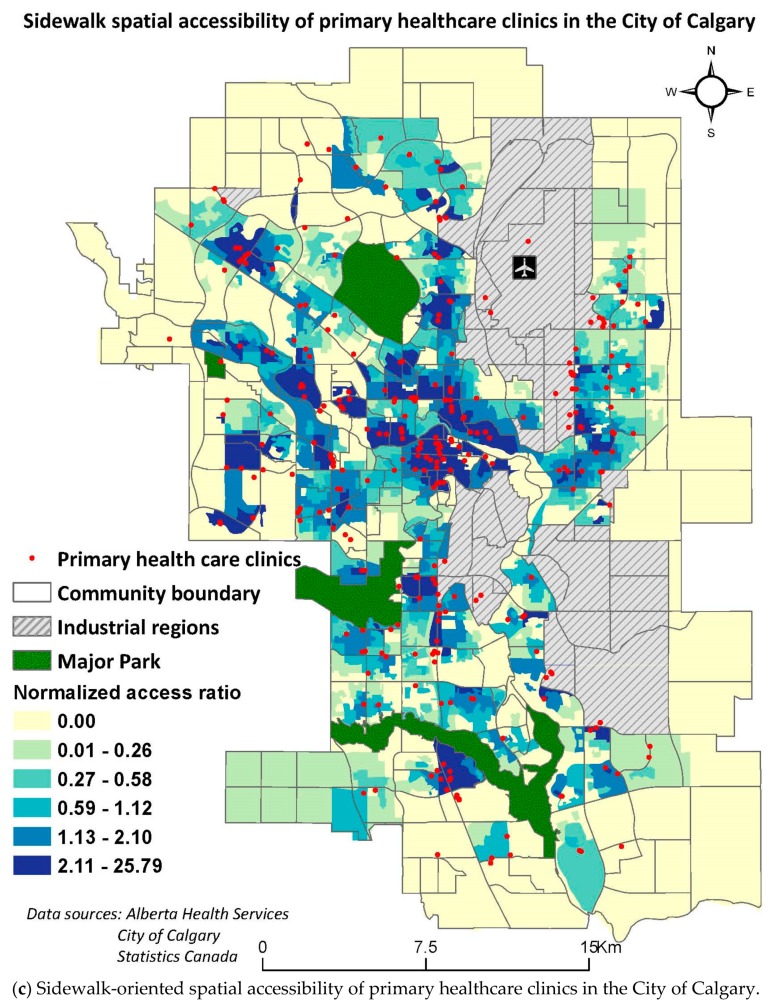
Spatial accessibility of the primary healthcare facilities in the City of Calgary by (**a**) driving, (**b**) multimodal means and (**c**) walking.

A general comparison between spatial accessibility trends by driving, multimodal means, and walking illustrate that there are higher accessibility values estimated in the urban (core) region as compared to suburban regions by all modes of travel (Figure 5, a: road, b: multimodal, and c: walking). Additionally, the regions of high spatial accessibility are identified at the locations of the healthcare clinics for all analyses. In other words, the regions which were determined to have no accessibility were found to contain no healthcare facility in their proximity. A general comparison in travel by different modes revealed that spatial accessibility decreases when the mode of travel is changed from car to bus transit means, and reduced further by walking.

### 3.1. Accessibility by Driving

The greatest spatial accessibility to primary healthcare facilities in the City of Calgary was achieved by driving as compared to other modes of travel (Figure 5a). The initial inspection of the access status through this mode points out the decreasing trend in accessibility as one moves from the central to the peripheral regions of the city. According to this model, precisely 24 out of 1594 DAs, with the cumulative population of 30,090 (2.4% of the total population), had no spatial accessibility to primary healthcare facilities. Most of the DAs with no accessibility were found to be located in the Northwest region of the city.

### 3.2. Accessibility by Multimodal Means of Travel (Sidewalks, Bus Routes and Train Lines)

As compared to driving mode, spatial accessibility by multimodal means exhibits more intra-community variations over the City of Calgary (Figure 5b). Through this mode, the access status was observed to lie between the accessibility measured by driving and walking networks (Figure 5a,c). Specifically, less intra-community variations are observed than measured through walking and higher intra-community variations are identified than analysis performed on driving. For the multimodal means of travel, 151 out of 1594 DAs were identified with no access to healthcare, which were dispersed in different sections of Calgary of cumulative population of 137,745 (11.1% of total population).

### 3.3. Accessibility by Walking

The spatial accessibility to the primary healthcare facilities in the City by walking (Figure 5c), is substantially lower than that of driving or multimodal means. For this analysis, prominent intra-community variations in accessibility indices are observed. This implies that the access is not the same within the communities by walking, contradictory to the results obtained by other means. For this travel mode, 455 out of 1594 DAs were identified with no access to primary healthcare, which is home to 439,500 (35.5%) people in the city. Unlike access measured by driving mode, the regions with no accessibility can be found in all four quadrants of the city. Generally, regions with higher access are concentrated in the downtown section of the city.

### 3.4. Multiple Regression Analysis by Mode of Travel

The regression analysis for three modes of travel resulted in positively spatially auto-correlated residuals, implied by the high positive values of the Moran’s I statistic (Table 4). This implies there is a spatial factor in determining the accessibility of primary healthcare facilities in the City of Calgary. This violated one of the assumptions of multiple regression. In other words, the residuals of the regression models should be independent of one another for the model to be considered valid. However, this was not the case in this research with positively auto-correlated residuals. Hence, the spatial regression analysis was performed to obtain regression results, while accounting for the spatial dependency of the nearby feature values.

For the spatial regression analysis for travel by car, four variables were determined to be significant with less than 0.05 *p*-value: proportion of the individuals separated, divorced, or widowed; proportion of the persons living alone; average income; and proportion of single-parent families (Table 5).

The global spatial regression model (Table 6) reveals that there is an inverse relationship between the spatial accessibility and the proportion of the individuals separated, divorced, or widowed. In other words, the regions with high proportion of separated, divorced, or widowed individuals have lower accessibility to primary healthcare. On the other hand, the variables which were found to have a positive relationship with the increased SPAR were found to be proportion of the persons living alone, average income, and the proportion of single-parent families. Specifically, there seems to be a mismatch between the needs and resources for primary healthcare provisions. People with higher income tend to make use of healthcare facilities more as compared to low income individuals. The possible explanations for these trends are presented in discussion.

In regards to the multimodal means analysis, variables identified to be significant were proportion of the individuals separated, divorced, or widowed; proportion of population living alone; average income; and employment-population ratio. According to the SPAR model, there is a direct relationship between the SPAR values and these variables except for proportion of the individuals separated, divorced, or widowed. As the regions of high proportion of population living alone, high average income, and high employment-population ratio increases, the SPAR value increases. On the other hand, the regions with high proportion of the individuals separated, divorced, or widowed were found to have the low SPAR values as seen in the driving mode of travel analysis. Again, possible explanations are provided in discussion.

When the mode of travel is changed to walking, the spatial regression results are different from the other two analyses. In this case, the significant variables were calculated to be proportion of the individuals separated, divorced, or widowed and proportion of people living alone. The trend between the SPAR values and these two variables is similar as observed for the SPAR analysis in other two modes of travel scenarios. The regions with high proportion of people living alone and low proportion of the individuals separated, divorced, or widowed were found in high spatial access areas (Table 7). These trends are further discussed in the discussion section.

## 4. Discussion

### 4.1. Accessibility Status of the Primary Healthcare Facilities by Different Modes of Travel

In regard to spatial accessibility measured by all three different modes, the regions which were identified to have no spatial accessibility were found to contain no healthcare facilities in its vicinity. This implies that the spatial distribution of primary healthcare facilities is non-uniform and hence, points out the spatial disparity in terms of healthcare allocation in space. A general comparison for accessibility to primary healthcare facilities with different modes revealed that populations without access to a car have a significantly lower access ratio as compared to population who can drive (Table 8). It is evident that both the shortage area and population served increased in number in the following order of travel mode considered: walking, multimodal (bus routes and train), and car. This implies that if the population does not have access to an automobile and relies on bus transit/train for mobility, the accessibility index of the travel to the primary healthcare facilities decreases; comparatively larger areas of the City of Calgary are found to have no accessibility to primary healthcare facilities. As a result, a higher proportion of the population of the City of Calgary is not served by the healthcare systems equally due to the distance barrier posed from limited access by public transit and walking.

Additionally, while comparing the results from different modes, it should be noted that the range of SPAR values differs among different modes. Specifically, the spatial accessibility range is lowest for the analysis by car travel (0.00–1.47). This analysis assumed that all of the population had access to a car, which resulted in the increasing ability to access the primary care overall. The small range implies that the assumption of universal access to a car smoothed the differences between the accessibility measures, resulting in the lower standard deviation (low variation in access in the City of Calgary). As the mode of travel is changed to multimodal (walking, bus routes, and train lines), the range increases (0.00–4.07). It can be deduced that more regional variability is identified with multi-modal network analysis as compared to the car analysis. This might have resulted from the kind of infrastructure in place that not all roads are bus routes, resulting in limiting choices of the primary healthcare facilities to the population to regions where bus service or the train lines are available. Another rationality behind this greater variation in access over space might be the unavailability of sidewalk infrastructure in non-core regions of the City of Calgary, resulting in limited access to the facilities. Further, the regional variability is highest for the walkability analysis (0.00–25.79). It is inferred that this would have resulted from the lower speed of pedestrians as compared to speeds in other travel models, concluding in higher spatial differences in the final output.

### 4.2. Relationship between Spatial Accessibility and Social Determinants of Health

The regression analysis between the accessibility index and the social determinants of health provided different results for each mode of travel. The aim of regression analysis was to detect any regions with low accessibility, where the vulnerable population is residing. There were two significant variables that were consistent among the three analyses: proportion of people living alone and proportion of the individuals separated, divorced, or widowed. This points out the mismatch between the needs and resources in primary healthcare provision. Specifically, it is important to determine the areas where there is a greater proportion of the individuals separated, divorced, or widowed, as these areas were found to have low access. Two key limitations were identified relating to the accuracy of travels speeds and actual car use, and employment status of the doctors. The same evaluation was used for all the features in travel networks; whereas, different elevation can result in travel at different speeds. Because of Calgary’s location in the foothills of the Rocky Mountains, the elevation varies from one place to another. We also did not consider time delays at intersections, leading to variations in travel speeds. Since the analyses were conducted on the travel time, not considering the elevation and delay times at intersections might not have truly captured the real-time spatial accessibility status of primary care in the city of Calgary. We also did not consider the status of access to a car through ownership, compared to new approaches to mobility such as car sharing, which in future studies, could be analyzed and paired with the access results. Another limitation is that the number of Full-Time Equivalent physicians, service hours, and days of clinics’ workings were not considered in the analysis. This information is crucial in determining whether the facility is capable of providing services. Ignoring these variables in the analysis might lead to inaccurate results regarding the availability of primary care in the city. There is also a lack of consideration of the general public’s perception on their accessibility status to primary healthcare facilities in this analysis. Qualitative data on the public’s perception of access and quantitative determination of access as performed in this analysis can be compared to obtain a holistic view of spatial accessibility to primary healthcare facilities in the City of Calgary.

Regardless of the limitations, it is important to consider the findings of this study to advocate for access for all populations regardless of socio-economic factors, such as access to a vehicle. The underlying purpose of this research was to examine the current situation of primary healthcare status in regards to clinic location, the population ability to access them, and the mode of travel used to travel to the healthcare services. An important next step would be to consider these factors to target future services to areas with the lowest (and in some cases nonexistent) access.

## 5. Conclusions

This research points out one of the biggest gaps in healthcare accessibility studies to date. The problem persists as most of the studies are conducted on the assumption of universal access to car. Specifically, in urban areas, a major proportion of people rely exclusively on public transportation for travel; serving as the motivation of this research. Specifically, we compared the effectiveness of different modes of travel in regard to accessibility to the primary healthcare facilities in the City of Calgary. People with access to a car were found to have the highest level of spatial accessibility to primary healthcare facilities in the city, with only 2.5% of the population in the shortage area. On the other hand, limited access was found for people relying on public transportation for accessing healthcare (11.1% of the population) and the lowest access was identified when the mode of travel was changed to walking only—over one third of the population (35.5%) reside in the shortage area. In other words, the social disparity in access to healthcare facilities was identified to be 14 times higher for people without access to a car. The regression analysis showed that the low-income regions corresponded to high access values. These were consistent with the previous research pointing out that people residing in low-income neighborhoods tend to utilize more healthcare services. Other variables were not consistent throughout different modes of travel analysis.

It is concluded that in the City of Calgary more primary healthcare facilities are required to be located in under-served areas or the pedestrian, or public transportation infrastructure needs to be improved—or ideally both. This study is important as it advocates for access without financial, environmental and ethical barriers as no person should have limited access to health due to not having an access to a private vehicle as everyone has an equal right to proper healthcare access. It can be deduced from the accessibility outputs that large portions of the City of Calgary have a low walking and public transit access. An effective solution to this problem might be to lessen the zoning restriction in certain communities to accommodate more primary healthcare facilities in the City of Calgary. Alternatively, walkability can be improved overall if the pedestrian infrastructure is enhanced in the City of Calgary. These changes are expected to improve the overall access to primary healthcare.

## Figures and Tables

**Table 1:** Study data.

Dataset	Year	Source
Primary healthcare clinics	2017	Alberta Health Services
Calgary dissemination areas (DA)	2016	Statistics Canada
Population weighted centroids	2016	Statistics Canada
Calgary communities	2016	Open Calgary
Sidewalks and trails	2014	City of Calgary
Pathways	2016	Open Calgary
Road network	2016	City of Calgary
Road intersections	2016	City of Calgary
Bus routes and stops	2017	City of Calgary
Train lines (C-train) & Stations	2017	City of Calgary

**Table 2:** Different road speed specifications.

Road Type	Speed (Km/h)
Collector	50
Major	65
Expressway	80
Alley	15

**Table 3:** Pampalon index variables.

Variables
Proportion of the individuals separated, divorced, or widowed
Proportion of the persons living alone
Proportion of single-parent families
Proportion of persons without a high school diploma
Employment-population ratio
Average income

**Table 4:** Spatial autocorrelation of residuals in DAs by different modes of travel.

Mode of Travel	Moran’s I	*p*-Value
Walking	0.54	2.20 × 10^−16^
Multimodal	0.46	2.20 × 10^−16^
Car	0.49	2.20 × 10^−16^

**Table 5:** Relationship between significant variables and accessibility index for travel by car.

Coefficients	Estimate	Std. Error	*z*-Value	*p*-Value
1. Intercept	2.34 × 10^−^^1^	1.58 × 10^−^^2^	14.8425	<2.20 × 10^−^^16^
2. Proportion of the individuals separated, divorced, or widowed	−2.30 × 10^−^^1^	8.22 × 10^−^^2^	−2.7953	0.005186
3. Proportion of the persons living alone	4.18 × 10^−^^1^	3.40 × 10^−^^2^	12.2891	<2.20 × 10^−^^16^
4. Average income	3.34 × 10^−^^7^	8.31 × 10^−^^8^	4.0139	5.97 × 10^−^^5^
5. Proportion of single-parent families	1.25 × 10^−^^1^	5.54 × 10^−^^2^	2.2541	0.024192
Rho: 0.66, LR test value: 1013.3, *p*-value: 2.22 × 10^−^^1^				
AIC: −1370.7				

Rho: Spatial autoregressive coefficient; LR: Lagrange Multiplier; AIC: Akaike Information Criterion.

**Table 6:** Relationship between significant variables and accessibility index for travel by multimodal means.

Coefficients	Estimate	Std. Error	*z*-Value	*p*-Value
1. Intercept	1.46 × 10^−^^1^	9.82 × 10^−^^2^	1.4835	0.13793
2. Proportion of the individuals separated, divorced, or widowed	−2.48 × 10^+0^	2.72 × 10^−^^1^	−9.1324	<2 × 10^−^^16^
3. Proportion of population living alone	1.80 × 10^+0^	1.24 × 10^−^^1^	14.583	<2 × 10^−^^16^
4. Average income	5.76 × 10^−^^7^	2.86 × 10^−^^7^	2.0162	0.04378
5. Employment-population ratio	2.46 × 10^−^^1^	1.37 × 10^−^^1^	1.7973	0.07229
Rho: 0.55485, LR test value: 668.09, *p*-value: <2.22 × 10^−^^16^				
AIC: 2463.6				

**Table 7:** Relationship between significant variables and accessibility index for travel by walking.

Coefficients	Estimate	Std. Error	*z*-Value	*p*-Value
1. Intercept	0.278341	0.077647	3.5847	0.000338
2. Proportion of the individuals separated, divorced, or widowed	−2.39588	0.671931	−3.5657	0.000363
3. Proportion of population living alone	2.06158	0.289785	7.1142	1.13 × 10^−^^12^
Rho: 0.54755, LR test value: 608.09, *p*-value: <2.22 × 10^−^^16^				
AIC: 5595.9				

**Table 8:** Physician shortage area statistics.

Mode of Travel	Shortage Area (km^2^)	Population in the Shortage Areas	% of Total Population in Shortage Area
Driving	50.6	30,090	2.5%
Multimodal	140.9	137,745	11.1%
Walking	520.9	439,500	35.5%
